# Comparison of cell-based assays for the identification and evaluation of competitive CXCR4 inhibitors

**DOI:** 10.1371/journal.pone.0176057

**Published:** 2017-04-14

**Authors:** Anneleen Van Hout, Thomas D’huys, Merel Oeyen, Dominique Schols, Tom Van Loy

**Affiliations:** Laboratory of Virology and Chemotherapy, Department of Microbiology and Immunology, Rega Institute for Medical Research, KU Leuven, Leuven, Belgium; Deutsches Primatenzentrum GmbH - Leibniz-Institut fur Primatenforschung, GERMANY

## Abstract

The chemokine receptor CXCR4 is activated by its unique chemokine ligand CXCL12 and regulates many physiological and developmental processes such as hematopoietic cell trafficking. CXCR4 is also one of the main co-receptors for human immunodeficiency virus (HIV) entry. Dysfunction of the CXCL12/CXCR4 axis contributes to several human pathologies, including cancer and inflammatory diseases. Consequently, inhibition of CXCR4 activation is recognized as an attractive target for therapeutic intervention. In this regard, numerous agents modifying CXCR4 activity have been evaluated in *in vitro* experimental studies and pre-clinical models. Here, we evaluated a CXCL12 competition binding assay for its potential as a valuable initial screen for functional and competitive CXCR4 inhibitors. In total, 11 structurally diverse compounds were included in a side-by-side comparison of *in vitro* CXCR4 cell-based assays, such as CXCL12 competition binding, CXCL12-induced calcium signaling, CXCR4 internalization, CXCL12-guided cell migration and CXCR4-specific HIV-1 replication experiments. Our data indicated that agents that inhibit CXCL12 binding, *i*.*e*. the anti-CXCR4 peptide analogs T22, T140 and TC14012 and the small molecule antagonists AMD3100, AMD3465, AMD11070 and IT1t showed inhibitory activity with consistent relative potencies in all further applied CXCR4-related assays. Accordingly, agents exerting no or very weak receptor binding (i.e., CTCE-9908, WZ811, Me6TREN and gambogic acid) showed no or very poor anti-CXCR4 inhibitory activity. Thus, CXCL12 competition binding studies were proven to be highly valuable as an initial screening assay and indicative for the pharmacological and functional profile of competitive CXCR4 antagonists, which will help the design of new potent CXCR4 inhibitors.

## Introduction

Human chemokines are chemotactic cytokines that are categorized based on the positioning of well-conserved N-terminal cysteine (C) residues and classified as C, CC, CXC, CX_3_C chemokines. By activating their corresponding chemokine receptors, that belong to the rhodopsin-like seven transmembrane G protein-coupled receptor (GPCR) family, chemokines play a pivotal role in many processes, including hematopoiesis, immune cell trafficking and stem and progenitor cell mobility [1]. Not surprisingly, aberrant chemokine receptor signaling underlies many human diseases, including multiple types of cancer [2, 3].

CXC chemokine receptor 4 (CXCR4) is widely expressed on hematopoietic cells [*e*.*g*. T- and B-lymphocytes, stem and progenitor cells (HSPCs)] as well as on endothelial and epithelial cells and in diverse tissues (*e*.*g*. lung, liver, colon and heart) [4]. Upon interaction with its unique chemokine ligand, CXC chemokine ligand 12 (CXCL12) [or stromal cell-derived factor-1 (SDF-1)], both G protein-dependent and–independent signaling pathways are activated along the CXCL12/CXCR4 axis, thereby regulating many biological responses, some of which can have severe clinical impact [5]. Together with CC chemokine receptor 5 (CCR5), CXCR4 is a major co-receptor facilitating human immunodeficiency virus (HIV) entry in cluster of differentiation 4 (CD4) positive T-lymphocytes [6, 7]. More recently, CXCR4 downstream signaling pathways have been shown to contribute to cancer progression by supporting the proliferation and survival of tumor cells and promoting tumor-related angiogenesis and metastasis of various types of human cancer [4, 8]. CXCR4 is also associated with several autoimmune diseases (*e*.*g*. rheumatoid arthritis) and warts, hypogammaglobulinemia, immunodeficiency, myelokathexis (WHIM) syndrome is caused by activating mutations in *cxcr4* [9, 10]. Taken together, interference with the CXCL12/CXCR4 axis might be of clinical significance for the treatment of various human diseases. In 2009, the first CXCR4 inhibitor [*i*.*e*. the small molecule AMD3100 (Plerixafor, Mozobil^®^)] was approved for clinical use to support the mobilization of hematopoietic progenitor cells required for stem cell transplantation in patients with hematological malignancies (*i*.*e*. myeloma and lymphoma) [11, 12].

Various CXCR4-targeting compounds, including anti-CXCR4 peptides, non-peptide small molecule antagonists, CXCL12 peptide analogs and anti-CXCR4 antibodies have so far been evaluated in *in vitro* experimental studies and pre-clinical animal models to investigate their effectiveness in inhibiting CXCR4 function [13, 14]. The variety of applied biological assays and experimental conditions, however, makes it difficult to truly compare their binding characteristics, relative potencies and mode of action (direct versus indirect mechanisms) which might hamper their use in novel disease models and further drug design. The goal of this study was thus to determine the binding characteristics of a selection of commercially available compounds previously claimed to inhibit *in vitro* and/or *in vivo* CXCR4 signaling and function and, in addition, to investigate to what extent these compounds are biased towards the inhibition of specific CXCR4-related pathways or functions. Therefore, all compounds were evaluated side-by-side in a panel of pharmacological and functional cell-based assays. In total, 11 compounds, the peptide analogs T22 [15], T140 [16], TC14012 [17] and CTCE-9908 [18] and the small molecules AMD3100 [12], AMD3465 [19], AMD11070 [20], IT1t [21], WZ811 [22], Me6TREN [23] and gambogic acid [24] were included, which were all initially tested for their ability to compete with CXCL12 at the level of receptor binding. In order to evaluate the potential bias towards a CXCR4-mediated signaling pathway or response, their activity was further evaluated in assays measuring their effect on CXCL12-induced calcium mobilization, CXCR4 internalization, CXCL12-guided chemotaxis and CXCR4-specific (X4) HIV entry and replication. A correlation between CXCR4 binding and the relative potency of compounds to inhibit CXCR4 signaling and function became apparent. In consequence, our data not only provide detailed insight into the CXCR4-inhibiting activity of commercially available compounds, but also demonstrate that applying CXCL12 competition binding studies can represent a highly informative step in future screening strategies for the identification of novel anti-CXCR4 compounds.

## Materials and methods

### Compounds, chemokines and antibodies

The structure of all used compounds is illustrated in S1 Fig. T22 [(Tyr^5.12^,Lys^7^)-Polyphemusin II; MW: 2,487.0 g/mol] [15] and T140 (MW: 2,037.5 g/mol) [16] were purchased from Bachem (Bubendorf, Switzerland). The compounds TC14012 (MW: 2,066.4 g/mol) [17], CTCE-9908 (MW: 1,927.3 g/mol) [18], IT1t (MW: 479.6 g/mol) [21] and AMD3465 Hexahydrobromide (MW: 896.1 g/mol) [19] were obtained from Tocris (Bristol, UK). Me6TREN (MW: 230.4 g/mol) [23] and gambogic acid (MW: 628.8 g/mol) [24] were ordered from Sigma-Aldrich (St. Louis, MO, USA). WZ811 (MW: 290.4 g/mol) [22] was purchased from Selleckchem (Houston, TX, USA). AMD11070 (MW: 349.5 g/mol) [20] and AMD3100 (MW: 830.5 g/mol) [12] were kindly provided by Dr. G. Bridger (at that time at AnorMed; Langley, Canada).

Recombinant human CXCL12 was obtained from Peprotech (Rocky Hill, NJ, USA). Alexa Fluor 647-labeled human CXCL12 (CXCL12^AF647^), which is synthesized by the incorporation of the AF647 conjugate (excitation maximum: 650 nm; emission maximum: 668 nm) at the C-terminal Lysine residue of CXCL12, was purchased from Almac (Craigavon, UK). The antibodies used in this study were phycoerythrin- (PE) and allophycocyanin (APC)-labeled mouse anti-human CXCR4 monoclonal antibodies (mAb) (clone 12G5; BD Pharmingen, San Diego, CA, USA), PE-conjugated rat anti-human CXCR4 mAb (clone 1D9; BD Pharmingen), PE-labeled mouse anti-human CXC chemokine receptor 7 (CXCR7) mAb (clone 10D1-J16; BioLegend, San Diego, CA, USA) and PE-conjugated mouse anti-human CD4 mAb (clone SK3; Biolegend) with the corresponding isotype controls [PE-labeled mouse IgG2a, κ isotype control mAb (clone G155-178; BD Pharmingen), APC-labeled mouse IgG2a, κ isotype control mAb (clone G155-178; BD Pharmingen), PE-conjugated rat IgG2a, κ isotype control mAb (clone R35-95; BD Pharmingen) and PE-labeled mouse IgG1, κ isotype control mAb (clone MOPC-21; BD Pharmingen)].

### Cell cultures

Jurkat and SUP-T1 cell lines were purchased from American type culture collection (ATCC, Manassas, VA, USA). The MT-4 cell line was a kind gift of Dr. L. Montagnier (at that time at the Pasteur Institute; Paris, France). CXCR4 surface expression on these cell lines was confirmed by flow cytometry (S2 Fig). These human T-lymphocytic cell lines were cultured in RPMI medium (Thermo Fisher Scientific, Waltham, MA, USA) containing 10% fetal bovine serum (FBS; Thermo Fisher Scientific) and 2 mM L-glutamine (Thermo Fisher Scientific). Human glioblastoma U87 cells, expressing CD4 (U87.CD4) or CD4 and CXCR4 (U87.CD4.CXCR4), were kindly provided by Dr. D. R. Littman (Skirball Institute of Biomolecular Medicine; NY, USA). U87.CD4.CXCR7 cells stably expressing human CXCR7 were constructed as described [25], from U87.CD4 cells transfected with pTEJ-8 plasmid DNA containing human CXCR7 cDNA. All glioblastoma cell lines were cultivated in Dulbecco’s modified eagle medium (DMEM; Thermo Fisher Scientific) supplemented with 10% FBS (Thermo Fisher Scientific), 0.01 M HEPES buffer (Thermo Fisher Scientific) and antibiotics [200 μg/ml geneticin (Thermo Fisher Scientific; CD4 selection) and 1 μg/ml puromycin (Sigma-Aldrich; CXCR4 and CXCR7 selection)]. CD4, CXCR4 and CXCR7 surface expression on these adherent cell lines was confirmed by flow cytometry throughout all experiments (S2 Fig and data not shown). Cell cultures were maintained at 37°C and 5% CO_2_ and subcultivated every two to three days.

Freshly isolated peripheral blood mononuclear cells (PBMCs) were isolated out of buffy coats from healthy donors (Red Cross, Belgium). They were cultured in RPMI medium with 10% FBS, 2 mM L-glutamine and stimulated with 2 μg/ml phytohemagglutinin (PHA; Sigma-Aldrich) for three days before use in the HIV replication assays (see below).

### Cellular cytotoxicity assay

Jurkat cells (3x10^5^ cells per well in culture medium) were incubated with serial dilutions of compound at room temperature (RT) for two hours. Cytotoxicity of the compounds was also evaluated at 37°C over a longer period of time in MT-4 cells (5x10^4^ cells per well in culture medium, four day incubation) and PHA-stimulated PBMCs (5x10^5^ cells per well in culture medium, ten day incubation) because these cell types were used in anti-HIV activity assays which last up to ten days (see below). Each condition was tested in duplicate. Cytotoxicity was evaluated microscopically and, in addition, cell viability was assessed using the colorimetric CellTiter 96 AQ_ueous_ One Solution Cell Proliferation Assay (Promega, Fitchburg, WI, USA), which is based on the reduction of the tetrazolium salt MTS [3-(4,5-dimethylthiazol-2-yl)-5-(3-carboxymethoxyphenyl)-2-(4-sulfophenyl)-2H-tetrazolium] to formazan by metabolically active cells. Absorbance at 490 nm was measured with the VersaMax ELISA Microplate Reader (Molecular Devices, Sunnyvale, CA, USA). The cytotoxic concentration 50 (CC_50_) of each compound was calculated based on the absorbance of negative (*i*.*e*. cells without compound) and positive (*i*.*e*. only culture medium) control samples.

### CXCL12^AF647^ binding assay

The CXCL12^AF647^ binding assay was performed according to Hatse *et al*. [26], but modified for use in a 96-well plate format. In brief, Jurkat cells were washed twice in assay buffer [Hank’s Balanced Salt Solution (HBSS; Thermo Fisher Scientific), 20 mM HEPES buffer (Thermo Fisher Scientific), 0.2% bovine serum albumin (Sigma-Aldrich), pH 7.4]. 3x10^5^ cells (in assay buffer) were incubated with test compound (in assay buffer) at RT for 15 minutes, followed by an additional incubation of 30 minutes with 2.9 nM CXCL12^AF647^. Non-treated Jurkat cells were incubated with the PE-labeled mouse anti-human CXCR4 mAb (clone 12G5) or PE-conjugated mouse IgG2a, κ isotype control mAb (clone G155-178) to verify CXCR4 expression levels. Afterwards, cells were washed twice in assay buffer and subsequently fixed in 1% paraformaldehyde in Dulbecco’s phosphate buffered saline (DPBS). Samples were analyzed with the FACSArray flow cytometer (Becton Dickinson, NJ, USA). Data were further analyzed with the FlowJo Software (Ashland, Oregon, USA). The percentage of CXCL12^AF647^ binding inhibition was calculated according to the formula [1 –(MFI_X_−MFI_NC_)/(MFI_PC_−MFI_NC_)] x 100, where MFI_X_ is the mean fluorescence intensity of the compound treated sample, MFI_NC_ the MFI of the negative control (*i*.*e*. autofluorescence of untreated and unlabeled cells) and MFI_PC_ the MFI of the positive control (*i*.*e*. cells exposed to CXCL12^AF647^ alone). The inhibitory concentration 50 (IC_50_; *i*.*e*. the compound concentration that inhibits CXCL12^AF647^ binding by 50%) was calculated for each compound.

### Calcium mobilization assay

Intracellular calcium fluxes were measured with the FLIPR Tetra system (excitation LED banks: 470–495 nm, emission filters: 515–575 nm; Molecular Devices). U87.CD4.CXCR4 cells were seeded at 2x10^4^ cells per well in gelatin-coated black-walled 96-well plates [0.1% gelatin in DPBS, incubated at RT for two hours and washed once with DPBS] and incubated at 37°C and 5% CO_2_ overnight. Then, cells were loaded with the fluorescent calcium indicator Fluo-2 acetoxymethyl ester (AM) (4 μM; excitation: 488 nm, emission: 515 nm; Sigma-Aldrich) dissolved (1:1 mixture) in pluronic acid solution (20% w/v; Sigma-Aldrich) at RT in the dark for 45 minutes. In parallel, a 96-well compound plate with different concentrations of compound, diluted in assay buffer (see above), and a chemokine plate with CXCL12 were prepared. Fluo-2 AM loaded cells were washed with assay buffer before being incubated in the FLIPR Tetra system at 37°C for five minutes. At the start of the kinetic measurement, compounds were added to the cell plate by the internal robotic system followed by an incubation period of 10 minutes during which changes in the intracellular calcium level were continuously measured by the FLIPR Tetra. Subsequently, CXCL12 was added (6.25 nM final concentration) and changes in cytosolic calcium concentration were further measured simultaneously in all 96 wells. Each condition was measured in triplicate and repeated several times as further indicated (see Results). For each sample, the difference between the maximum and minimum percentage of baseline (*i*.*e*. mean relative light units in each well during a fixed time interval before compound or CXCL12 addition) was calculated with the ScreenWorks 4.0^™^ software (Molecular Devices) and data were further processed using GraphPad Prism 7. IC_50_ value of each compound was determined based on negative (*i*.*e*. untreated cells without CXCL12 stimulation) and positive (*i*.*e*. untreated cells with CXCL12 stimulation) control samples.

### Internalization assay

T-lymphoblastic SUP-T1 cells were washed twice in DPBS containing 2% FBS. Afterwards, cells were first incubated with the PE-labeled rat anti-human CXCR4 mAb (clone 1D9; 2 μg/ml in DPBS/2% FBS; BD Pharmingen) on ice for 45 minutes. To assess aspecific binding, cells were also stained with the PE-conjugated rat IgG2a, κ isotype control mAb (clone R35-95; 2 μg/ml in DPBS/2% FBS; BD Pharmingen). SUP-T1 cells were subsequently washed once with ice cold DPBS and twice with ice cold assay buffer (see above) before being treated with test compound on ice for 15 minutes. Next, cells were stimulated with 6.25 nM CXCL12 at 37°C for 30 minutes to induce receptor internalization and transferred to an acidic DPBS solution (pH 2), to allow cleavage of all remaining, non-internalized cell surface CXCR4 receptors. To evaluate the potential agonistic activity of the compounds, compound-treated samples were incubated at 37°C in the absence of CXCL12. Samples were immediately analyzed with the FACSCalibur flow cytometer (Becton Dickinson) whereby the measured fluorescence corresponds to the amount of internalized CXCR4 receptors. Data were further analyzed with the FlowJo software. The IC_50_ (*i*.*e*. the compound concentration that inhibits the CXCL12-induced CXCR4 internalization by 50%) was calculated for each compound based on the MFI of the negative (*i*.*e*. cells without compound and not stimulated with CXCL12) and positive (*i*.*e*. cells without compound and stimulated with CXCL12) control.

### Chemotaxis assay

Jurkat cells were washed twice in assay buffer (see above), dispensed into a 96-well plate (5x10^5^ cells per well) containing serial dilutions of compounds (in assay buffer) and incubated at RT in the dark for 10 minutes. Meanwhile, 6.25 nM CXCL12 (in assay buffer) was added to the bottom wells of a 96-well chemotaxis plate (Corning, NY, USA). Suspensions of 1.87x10^4^ cells were transferred to the chemotaxis insert plate. Cells were allowed to migrate in response to CXCL12 at 37°C and 5% CO_2_ for two hours. After removal of the insert plate, migrated cells were centrifuged and resuspended in DPBS containing flow cytometry cell counting beads (SPHERO AccuCount Fluorescent Particles; Gentaur, London, UK). Both migrated cells and particles were counted by flow cytometry (FACSCanto II; Becton Dickinson). Each compound concentration was tested in triplicate. Data were analyzed using the FlowJo software and the total number of migrated cells was calculated, according to the counted and total number of beads present in the sample. IC_50_ values of the compounds were calculated relative to the total migrated cells in the negative (untreated, spontaneously migrated cells) and positive (untreated, cells migrated towards CXCL12) control samples.

### Anti-HIV activity assays

The anti-HIV replication assays in MT-4 cells and PHA-stimulated PBMCs have been described previously [27]. The HIV-1 T-tropic molecular clone NL4-3 (X4) was obtained from the National Institute of Allergy and Infectious Disease (NIAID) AIDS reagent program (Bethesda, MD, USA). Briefly, 5x10^4^ MT-4 cells (in cell culture medium) were treated with different concentrations of compound at 37°C for 30 minutes. Afterwards, a 50% tissue culture infectious dose of the NL4-3 viral stock was added to the cell-compound mixtures. Four days post infection, the cytopathic effect was evaluated microscopically and cell viability was assessed with the MTS method, as described above.

PHA-stimulated PBMCs at 5x10^5^ cells per sample, pre-incubated with test compounds dissolved in cell culture medium containing IL-2 (2 ng/ml; R&D systems Europe, Abingdon, UK), were infected with NL4-3. After three days, fresh culture medium with IL-2 was added. Ten days post infection cell supernatant was collected for HIV-1 p24 core antigen enzyme-linked immunosorbent assay (ELISA; Perkin Elmer, Boston, MA, USA) according to the manufacturer’s protocol.

In the antiviral assays, each condition was tested in duplicate. Based on the absorbance measured in the negative (*i*.*e*. untreated and uninfected cells) and positive (*i*.*e*. untreated virus-infected cells) control samples, the IC_50_, defined as the test compound concentration that inhibits HIV-induced cell death by 50%, was calculated.

## Results

### Cytotoxicity of the investigated compounds

In total, 11 compounds were included in this study: the CXCR4 peptide analogs T22 [15] and its derivatives (T140 [16] and TC14012 [17]), the CXCL12 peptide analog CTCE-9908 [18] and the non-peptide small molecule CXCR4 antagonists AMD3100 [12] and derivatives (AMD3465 [19], AMD11070 [20]), IT1t [21], WZ811 [22], Me6TREN [23] and gambogic acid [24] (S1 Fig). To determine the maximum compound concentration to be used in our panel of screening assays, the cellular cytotoxicity of all compounds was first evaluated. Based upon microscopical evaluation of Jurkat cells and quantification of cell viability using MTS, the cytotoxic concentration 50 (*i*.*e*. CC_50_; the concentration that reduces cell viability by 50%) was determined. CC_50_ values were ≥ 100 μM for all compounds, with exception of gambogic acid for which a CC_50_ value of 10 μM was obtained. In consequence, the potential anti-CXCR4 activity of gambogic acid was further assayed at concentrations up to 10 μM whereas for all other compounds 100 μM was used as the highest concentration. Similar results were obtained in MT-4 cells and PHA-stimulated PBMCs after four and ten days incubation with compounds, respectively. None of the compounds showed remarkable cytotoxicity in these cell types. Only gambogic acid was toxic in both MT-4 cells and PHA-stimulated PBMCs at low micromolar concentrations (CC_50_ ~ 300 nM).

### Effect of CXCR4 compounds on CXCL12 binding to CXCR4

**Fig 1:**
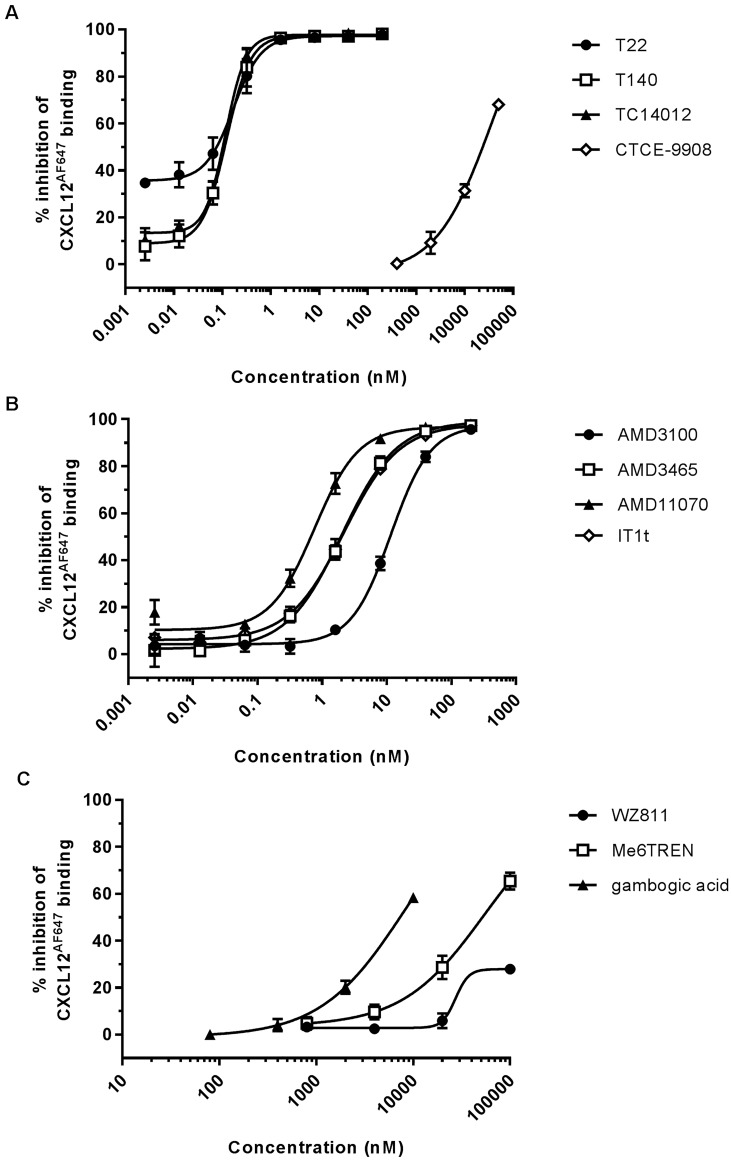
Interference with CXCL12/CXCR4 interaction by the tested compounds. (A), (B) and (C): Inhibition of the CXCL12/CXCR4 interaction by (A) the peptide analogs T22, T140, TC14012 and CTCE-9908 and the small molecules (B) AMD3100, AMD3465, AMD11070, IT1t and (C) WZ811, Me6TREN and gambogic acid. Jurkat cells were incubated with 2.9 nM CXCL12^AF647^ in the presence of different concentrations of compound. Data are represented as % inhibition of maximal CXCL12 binding to CXCR4. Mean ± SEM of three or four independent experiments is shown.

We investigated to what extent the different classes of agents were able to compete for receptor binding with CXCL12, the endogenous CXCR4 ligand. To ensure binding specificity, these experiments were performed using Jurkat cells endogenously expressing CXCR4, but not CXCR7, which shares CXCL12 as a common ligand (S2 Fig). Jurkat cells were pretreated with serial dilutions of compound and thereafter incubated with CXCL12^AF647^. The fluorescent signal of specific CXCL12^AF647^ binding was then quantified by flow cytometry. All compounds inhibited the CXCL12/CXCR4 interaction, albeit with different potencies (Fig 1; S1 Table). The peptide analogs T22, T140 and TC14012 and the small molecule antagonists AMD3100, AMD3465, AMD11070 and IT1t showed very potent and dose-dependent inhibition of the CXCL12/CXCR4 interaction with IC_50_ values in the low nanomolar range [IC_50_s: 0.079 ± 0.034 nM (T22), 0.12 ± 0.025 nM (T140), 0.11 ± 0.0094 nM (TC14012), 12.0 ± 1.1 nM (AMD3100), 2.1 ± 0.24 nM (AMD3465), 0.67 ± 0.10 nM (AMD11070), 2.1 ± 0.37 nM (IT1t)] (Fig 1A and 1B). In contrast, CTCE-9908, WZ811, Me6TREN and gambogic acid only weakly inhibited CXCL12 receptor binding with relatively high IC_50_ values in the micromolar range [IC_50_s: 19,145 ± 2,380 nM (CTCE-9908); >100,000 nM (WZ811); 51,934 ± 8,952 nM (Me6TREN); 7,063 ± 532 nM (gambogic acid)] (Fig 1A and 1C). In addition, all compounds were tested for their ability to inhibit CXCL12 binding on CXCR7-transfected U87 cells (U87.CD4.CXCR7) whereby only AMD11070 [28] and TC14012 [29] reduced CXCL12 binding on CXCR7 in the lower micromolar range, while all other compounds did not or only very weakly inhibit CXCL12/CXCR7 interaction (data not shown).

### Effect of CXCR4 compounds on CXCL12-induced intracellular calcium signaling

**Fig 2:**
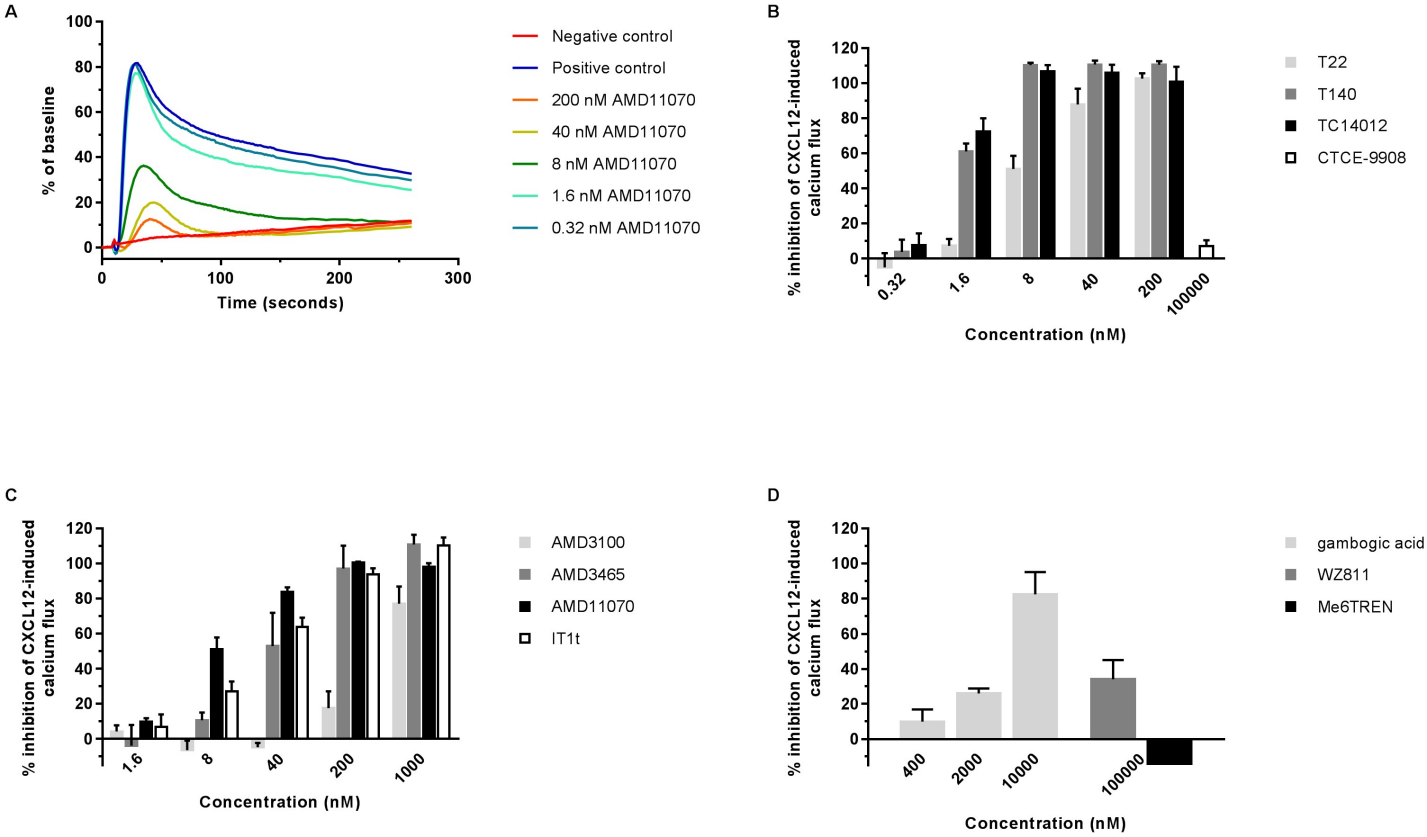
Antagonistic effect of compounds on CXCL12-induced calcium signaling in U87.CD4.CXCR4 cells. Fluo-2 AM loaded cells were treated with serial dilutions of compound and subsequently stimulated with 6.25 nM CXCL12. Fluorescence changes were measured over time in all wells simultaneously. (A): The inhibitory effect of different concentrations of AMD11070 on CXCL12-induced calcium release is shown. The red and blue line represent the negative (untreated, without CXCL12 stimulation) and positive (untreated, CXCL12 stimulation) control, respectively. Calcium fluxes are represented as % of baseline (*i*.*e*. mean relative light units in each well during a fixed time interval before CXCL12 addition). One representative experiment out of three is shown with each data point corresponding to the mean fluorescence of three to six replicates. (B), (C) and (D): Inhibitory effect of (B) T22, T140, TC14012, CTCE-9908, (C) AMD3100, AMD3465, AMD11070, IT1t, (D) WZ811, Me6TREN and gambogic acid on CXCL12-induced calcium flux. Data are represented as % inhibition of CXCL12-induced calcium mobilization relative to the positive and negative control. Mean ± SEM of three independent experiments is shown.

CXCL12 binding to the receptor will result in receptor activation. The transient increase in cytosolic calcium concentration is an important signaling pathway that is activated upon CXCR4 stimulation. The calcium mobilization assay allowed to evaluate both the potential agonistic (in absence of CXCL12) as well as the antagonistic activity (*i*.*e*. the potency to inhibit the CXCL12-induced calcium mobilization) of the compounds in real time using the FLIPR Tetra system (Fig 2; S3 Fig; S1 Table). U87.CD4.CXCR4 cells were first loaded with the fluorescent calcium indicator Fluo-2 AM. Treatment of cells with CXCL12 alone elicited a dose-dependent increase of the calcium concentration (EC_50_ = 1.6 nM, EC_90_ = 10.9 nM). This CXCL12-induced calcium flux was CXCR4 specific, since CXCL12 did not elicit a calcium flux in U87.CD4 cells (data not shown). The effect of the compounds on the intracellular calcium mobilization was determined by pre-incubating the cells with compound (agonistic activity; S3 Fig) and subsequently stimulating them with 6.25 nM CXCL12 (antagonistic activity; Fig 2). CTCE-9908 showed very weak agonist activity when tested at 100 μM (S3 Fig), and this activity was lost when tested at 20 μM. None of the other compounds displayed agonistic activity by themselves at the concentrations tested (S3 Fig). AMD11070 showed antagonistic activity as it dose-dependently inhibited the CXCL12-induced intracellular calcium flux (IC_50_: 12.3 ± 1.7 nM) (Fig 2A). This calcium flux was also inhibited by the peptide analogs T22, T140 and TC14012 and the low molecular weight antagonists AMD3100, AMD3465 and IT1t with the following IC_50_s: 9.3 ± 3.2 nM; 1.2 ± 0.17 nM; 0.95 ± 0.18 nM; 723.0 ± 99.1 nM; 53.4 ± 24.3 nM and 23.1 ± 4.6 nM, respectively (Fig 2B and 2C). Gambogic acid inhibited the calcium flux to a somewhat lesser extent (IC_50_: 4,371 ± 672 nM) (Fig 2D). 100 μM of CTCE-9908, WZ811 and Me6TREN did not affect the calcium flux evoked by CXCL12 (IC_50_s >100,000 nM) (Fig 2B and 2D).

### Inhibition of CXCL12-induced CXCR4 receptor internalization

**Fig 3:**
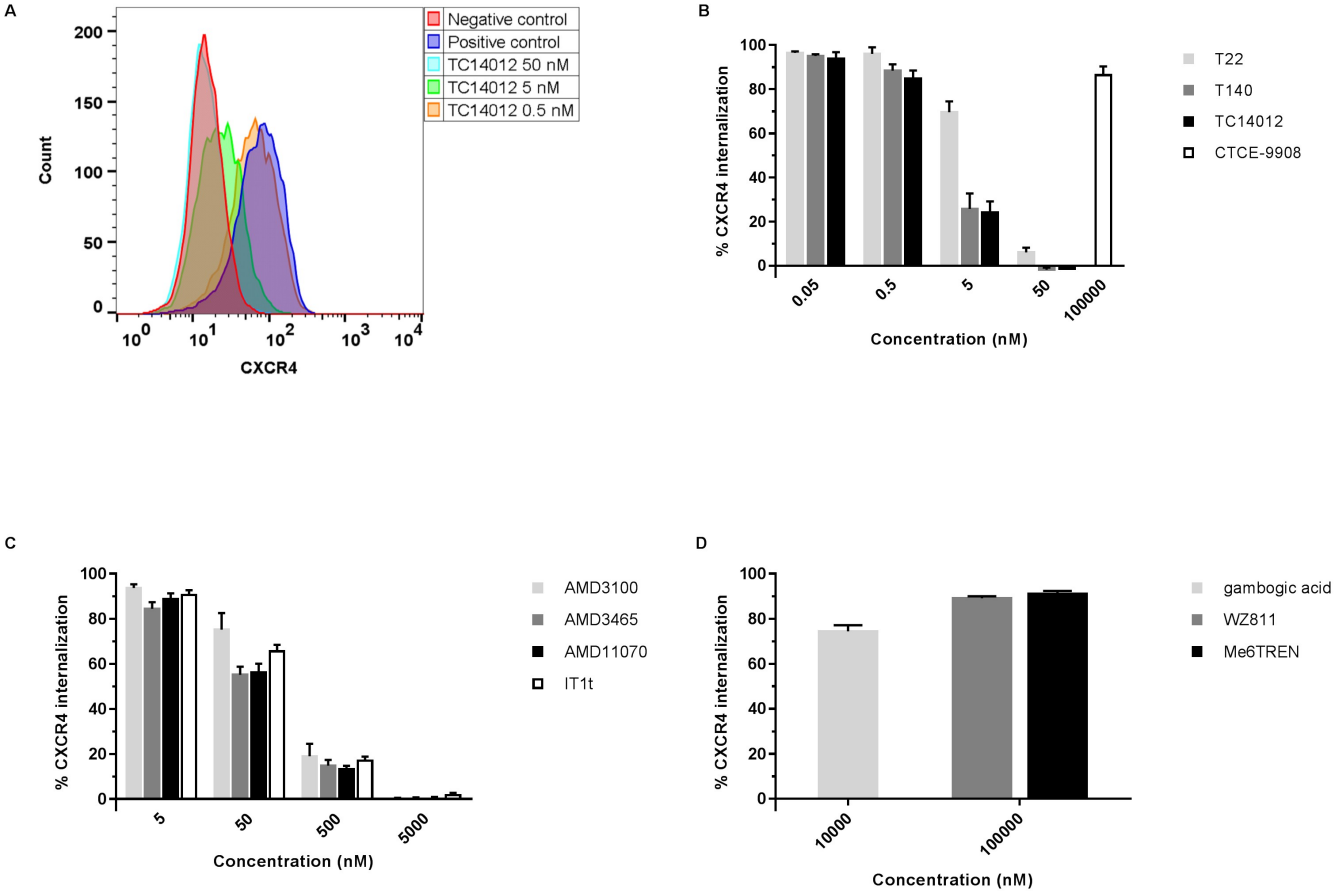
Inhibitory effect of CXCR4 compounds on CXCL12-induced receptor endocytosis in SUP-T1 cells. SUP-T1 cells were stained with a mAb targeting the N-terminus of CXCR4 (clone 1D9) and then treated with different concentrations of compound. CXCR4 internalization was induced by stimulating samples with CXCL12 at 37°C and quantified by flow cytometry. (A): Flow cytometric analysis of the negative (untreated, no CXCL12 stimulation; red histogram) and positive (untreated, CXCL12 stimulation; dark blue histogram) control and TC14012-treated samples (50 nM to 0.5 nM; light blue, green and orange histogram, respectively). (B), (C) and (D): Inhibitory effect of (B) T22, T140, TC14012, CTCE-9908, (C) AMD3100, AMD3465, AMD11070, IT1t, (D) WZ811, Me6TREN and gambogic acid on CXCR4 endocytosis. Data are represented as % of CXCR4 internalization relative to the positive and negative control. Mean ± SEM of at least two independent experiments is shown.

A feature of CXCR4, shared with many other GPCRs, is its internalization following agonist binding, also known as receptor endocytosis. To evaluate the potential inhibitory effect of the compounds on CXCR4 endocytosis, CXCR4 receptors endogenously expressed on SUP-T1 cells were first stained with a PE-labeled rat anti-human CXCR4 mAb targeting the receptor’s N-terminus (clone 1D9) and subsequently incubated with compound. Afterwards, samples were stimulated with CXCL12 to induce the sequestration of CXCR4 and the degree of receptor internalization was quantified by flow cytometry (Fig 3A). Control experiments in which stained SUP-T1 cells were stimulated with CXCL12 in absence of compound showed strong cellular staining (Fig 3A), thus indicating that CXCL12 did not compete with the labeled CXCR4 mAb for CXCR4 binding. In addition, when SUP-T1 cells were incubated with compound for thirty minutes at 4°C, and afterwards stained with the CXCR4 mAb (1D9) for thirty minutes at 4°C, no displacement (*i*.*e*. decrease in mAb binding) of the CXCR4 mAb was observed by flow cytometry (data not shown). This demonstrates that also the compounds themselves were unable to reduce mAb (1D9) binding to CXCR4. In consequence, reduced cellular staining in our assay reflects the inhibitory effects of compounds on receptor internalization and not simply displacement of the CXCR4 mAb.

No inhibition of CXCR4 internalization was observed when SUP-T1 cells were treated with CTCE-9908, WZ811, Me6TREN and gambogic acid at the tested concentrations (WZ811, Me6TREN and CTCE-9908: IC_50_s >100,000 nM; gambogic acid: IC_50_ >10,000 nM) (Fig 3B and 3D; S1 Table). All other compounds inhibited the CXCL12-induced CXCR4 internalization in a dose-dependent manner. T22 and derivatives were more potent inhibitors of CXCR4 internalization compared to the low molecular weight compounds AMD3100, AMD3465, AMD11070 and IT1t (Fig 3B and 3C). The following IC_50_s were obtained: 10.2 ± 1.5 nM (T22); 2.2 ± 0.4 nM (T140); 1.9 ± 0.2 nM (TC14012); 148.0 ± 36.0 nM (AMD3100); 67.3 ± 11.3 nM (AMD3465); 70.5 ± 13.1 nM (AMD11070) and 105.7 ± 12.3 nM (IT1t) (S1 Table). Of note, in absence of CXCL12, none of the tested compounds induced CXCR4 internalization at the highest concentration tested (*i*.*e*. 50 μM for T22, T140, TC14012, AMD3100, AMD3465, AMD11070 and IT1t; 100 μM for CTCE-9908, WZ811 and Me6TREN; 10 μM for gambogic acid), demonstrating the absence of an agonistic effect of the compounds themselves (data not shown).

### Antagonistic effects on CXCL12-directed migration of Jurkat cells

**Fig 4:**
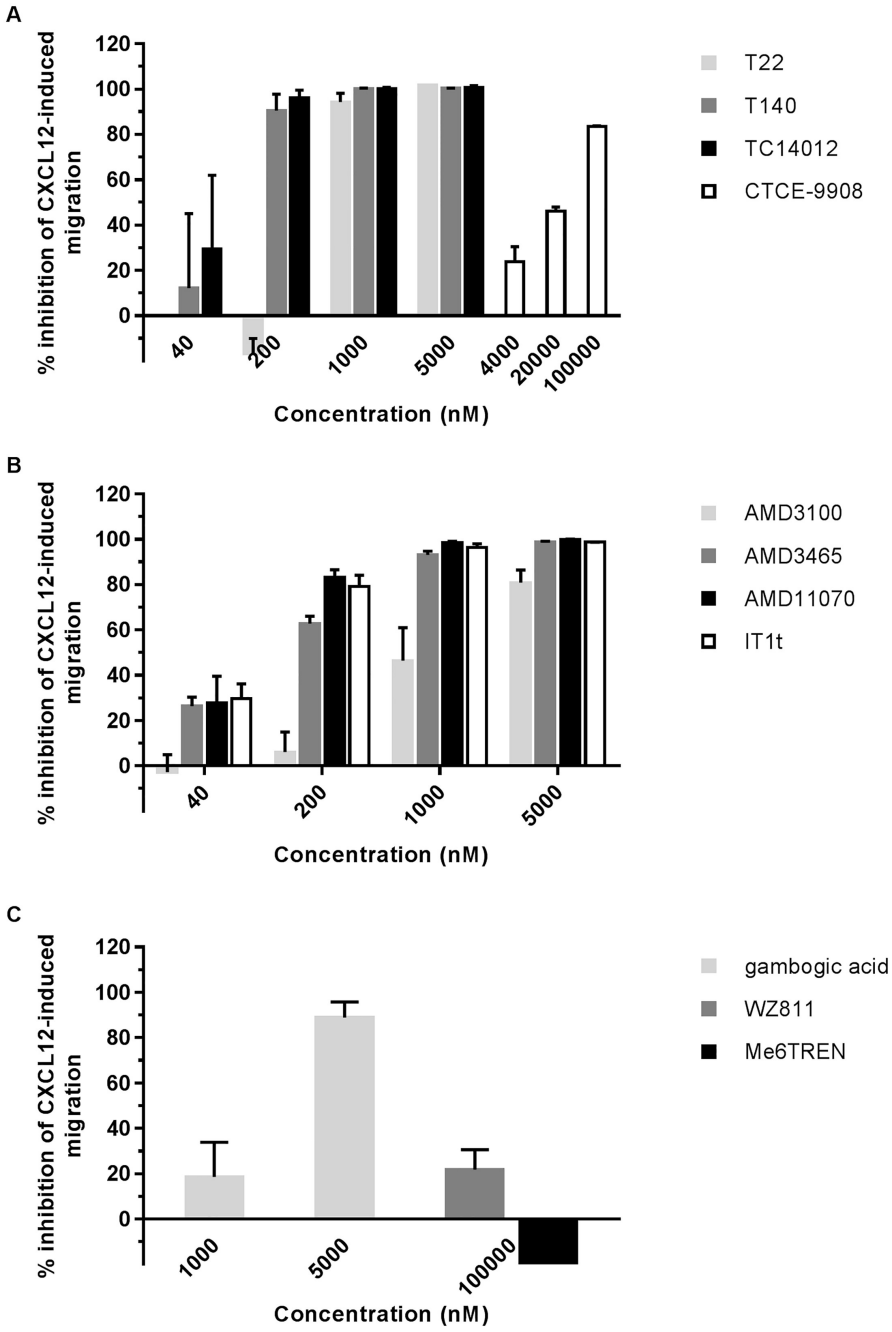
CXCL12-guided migration of Jurkat cells is inhibited by CXCR4-interacting compounds. (A), (B) and (C): Effect of (A) T22, T140, TC14012, CTCE-9908, (B) AMD3100, AMD3465, AMD11070, IT1t, (C) WZ811, Me6TREN and gambogic acid on chemotaxis of CXCR4-positive Jurkat cells. After compound treatment, cells were allowed to migrate towards 6.25 nM CXCL12, present in the lower compartment of the insert, for two hours. The migrated cells were counted by flow cytometry. Data are represented as % inhibition of migration relative to the negative and positive control (spontaneously migrated cells and cells migrated towards CXCL12, respectively). ± SEM of two to three independent experiments, each performed in triplicate.

The directional migration of leukocytes and stem and progenitor cells is a key function of chemokines. CXCL12 binding to CXCR4-expressing cells will evoke a chemotactic biological effect on these cells. The capacity of the compounds to inhibit the chemotaxis of Jurkat cells was determined using transwell migration assays (Fig 4; S1 Table). CXCL12 at 6.25 nM elicited a strong chemotactic response on CXCR4-positive Jurkat cells (positive control) compared to buffer-treated cells (negative control). WZ811 and Me6TREN, tested at 100 μM, did not inhibit the migration of Jurkat cells (IC_50_s >100,000 nM) (Fig 4C). CTCE-9908 and gambogic acid were very weak inhibitors of migration (with the following IC_50_s,: 23,593 ± 1,711 nM and 1,888 ± 353 nM, respectively), while a potent and dose-dependent inhibitory effect of chemotaxis by IT1t, AMD3100 and T22 and its derivatives was observed [IC_50_s: 470.2 ± 16.5 nM (T22); 73.7 ± 26.0 nM (T140); 58.3 ± 22.3 nM (TC14012); 1,431 ± 485 nM (AMD3100); 116.0 ± 15.2 nM (AMD3465); 74.9 ± 17.7 nM (AMD11070) and 79.1 ± 9.6 nM (IT1t)] (Fig 4). The chemotactic response of Jurkat cells was most effectively inhibited by TC14012. A total inhibition of migration was obtained when cells were pretreated with TC14012 at concentrations as low as 200 nM (Fig 4A).

### Evaluation of the anti-HIV activity against X4 HIV-1 NL4-3

A key role for CXCR4 is its involvement as a co-receptor in HIV viral entry [6]. The antiviral activity of the compounds against the laboratory X4-tropic HIV-1 strain NL4-3 was determined in MT-4 cells and PHA-stimulated PBMCs. Cells were pretreated with non-toxic compound concentrations and subsequently infected with HIV-1 NL4-3. Viral replication was quantified four days (MT-4 cells; MTS assay) or ten days (PHA-stimulated PBMCs; p24 Ag ELISA) post infection. Similar antiviral activity of the compounds was observed in both cell types. CTCE-9908, WZ811, Me6TREN and gambogic acid were not or very weakly active against HIV-1 replication when tested at nontoxic concentrations. TC14012 was an improved inhibitor of viral replication compared to the two other polyphemusin II-derived peptides T22 and T140. The antiviral potency of the AMD inhibitors AMD3100, AMD3465 and AMD11070 was comparable in both MT-4 cells and PHA-stimulated PBMCs (Table 1).

**Table 1:** Anti-HIV-1 (X4) activity of CXCR4 compounds evaluated in CD4-positive MT-4 cells and PHA-stimulated PBMCs.

	IC_50_^a^ (nM)	
	MT-4 cells	PHA-stimulated PBMCs
T22	96.0 ± 10.5	64.0 ± 12.1
T140	13.5 ± 7.1	10.4 ± 4.8
TC14012	7.2 ± 2.3	9.3 ± 4.0
CTCE-9908	16,499 ± 1,985	59,568 ± 11,142
AMD3100	11.8 ± 3.4	14.9 ± 3.2
AMD3465	21.8 ± 10.1	27.2 ± 8.7
AMD11070	11.9 ± 4.6	26.4 ± 6.1
IT1t	14.2 ± 5.3	19.0 ± 2.3
WZ811	>100,000	38,234 ± 3,794
Me6TREN	>100,000	>100,000
gambogic acid	>376.1	>225.2

^a^ Compound concentration required to inhibit HIV-1 (X4) NL4-3 replication by 50%. Mean IC_50_ ± SEM are represented of four (MT-4 cells) or two to four (PHA-stimulated PBMCs) separate experiments.

**Fig 5:**
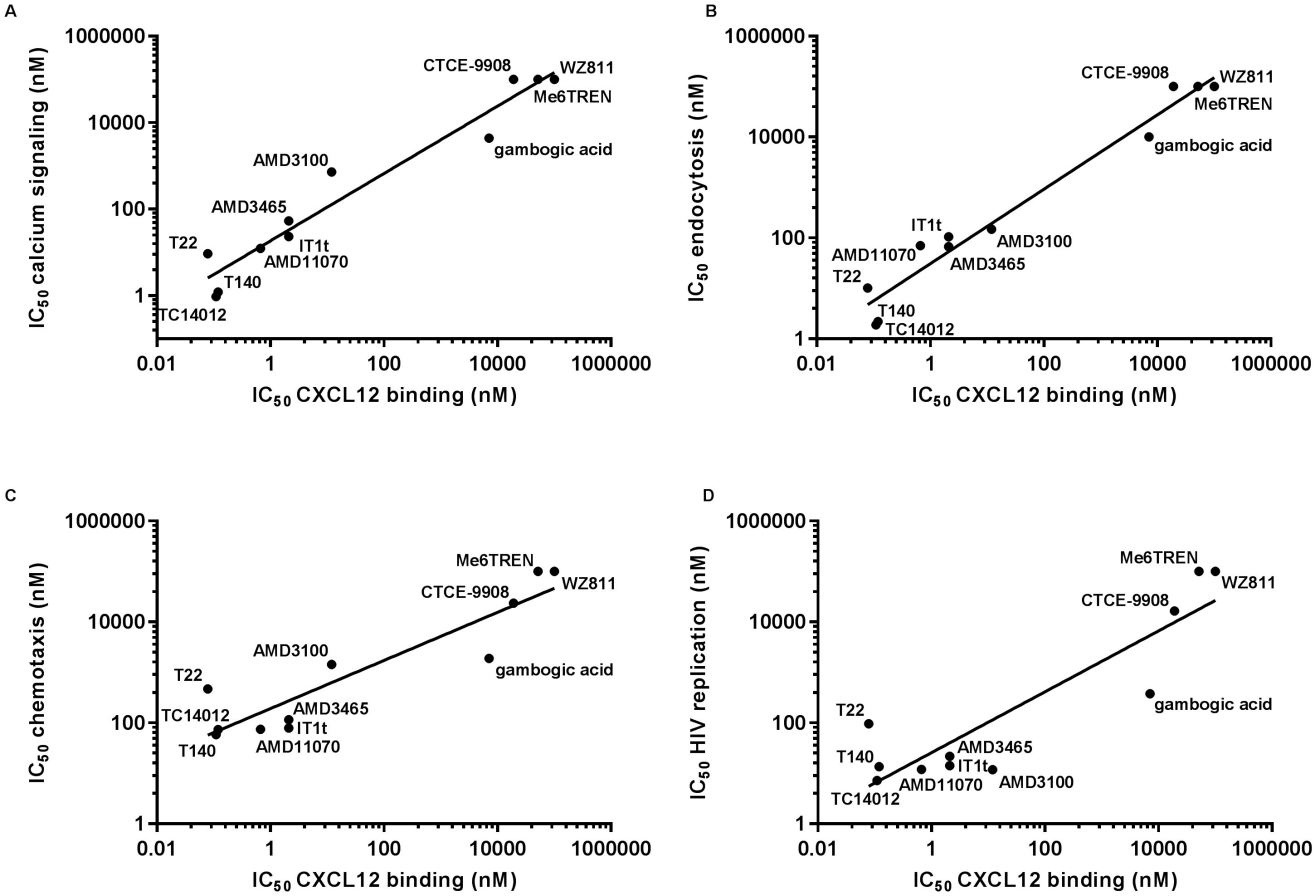
Correlation between the CXCR4 binding characteristics of the compounds and their potency to inhibit CXCL12/CXCR4 signaling and function. **(A), (B), (C) and (D):** Correlation between the IC_50_ value of the compounds for CXCL12 receptor binding inhibition and inhibition of (A) CXCL12-induced calcium signaling (p<0.0001; R^2^ = 0.95), (B) CXCR4 internalization (p<0.0001; R^2^ = 0.96), (C) chemotaxis (p<0.0001; R^2^ = 0.84) and (D) HIV viral entry (in MT-4 cells) (p = 0.0002; R^2^ = 0.81). The maximum test concentration of CTCE-9908, WZ811, Me6TREN and gambogic acid is represented when no activity was detected. Correlations were calculated in Graphpad using the Pearson correlation test (p-values < 0.05 were considered as statistical significant).

Taken together, our results highlight a correlation between the activity of the compounds to inhibit CXCL12 receptor binding on the one hand, and calcium mobilization (Fig 5A), CXCR4 receptor internalization (Fig 5B), cell migration (Fig 5C) and HIV entry (Fig 5D) on the other hand. This indicates that CXCL12 binding experiments are very informative as an initial screening assay to evaluate the activity of competitive CXCR4 inhibitors.

## Discussion

Given the early discovery that chemokine receptor CXCR4 facilitates HIV-1 entry in CD4-positive T-lymphocytes, this receptor was initially studied as a candidate drug target for anti-HIV therapy [6, 14]. This led to the discovery of several CXCR4-targeting compounds capable of inhibiting HIV infection in CD4-positive T-lymphocytes, including the cyclams, polyphemusin II and analogs derived thereof. Due to growing evidence that supports an important role for CXCR4 in tumor progression, stem and progenitor cell mobility and autoimmune diseases, CXCR4 gained broad attention as an attractive target for therapeutic intervention in various disease areas. Multiple strategies for inhibiting CXCR4 function, including small molecule antagonists, peptide inhibitors, neutralizing antibodies and nanobodies have been developed and evaluated in *in vitro* assays and *in vivo* pre-clinical models [13]. Due to the divergent signaling pathways evoked upon CXCR4 stimulation, a multitude of screening and functional assays can be envisaged for drug discovery. To our knowledge, this study presents for the first time a side-by-side comparative evaluation of commercially available CXCR4-targeting agents (S1 Fig) in diverse cell-based assays, which until now has been hampered by the divergence of experimental conditions used in previous studies.

Our work suggests that profiling the compound’s binding characteristics via competition binding studies with the orthosteric ligand (*i*.*e*. CXCL12) is predictive for its general pharmacological and functional anti-CXCR4 activity. Hence, evaluating the binding characteristics of novel compounds compared to a given reference compound might be a valuable initial screening strategy guiding novel drug discovery workflows in order to evaluate the potency of competitive CXCR4 antagonists. Until now, it has also been unclear if the compounds included in this study show a bias towards the inhibition of a particular pathway or functional response. Here, we demonstrate that this is likely not the case. A compound shown to physically interact with the ligand binding pocket of CXCR4 effectively inhibited the response in all signal transduction and functional receptor assays tested with consistent relative potencies, while compounds that did not interfere with agonist binding showed no or only very limited effect (Fig 5). Biased GPCR ligands, including antagonists that preferentially inhibit one functional or signaling pathway over the other have been described for other GPCRs [30, 31] and raise general interest since they might evoke less side effects in clinical settings [32]. Our data, however, indicate that compounds commonly used to interrupt CXCL12/CXCR4 interaction display no bias in activity when tested in functional assays relevant for CXCR4. Obviously, this does not exclude that biased CXCR4 antagonists, or biased agonists that stabilize particular active receptor conformations while blocking others, might be discovered in the future.

Several studies have previously reported *in vivo* inhibitory activity of Me6TREN [23] and CTCE-9908 [18] without demonstration of a direct interaction with the CXCR4 receptor. Our data confirm this lack of a direct effect on CXCR4 and thus imply that CXCR4 function can still be downregulated *in vivo* without the requirement for a direct receptor-compound interaction. It has been suggested that the small molecule Me6TREN mobilizes *in vivo* HSPCs by upregulation of matrix metallopeptidase-9 (MMP-9), which subsequently decreases CXCL12 levels in the bone marrow, leading to reduced CXCL12/CXCR4 interaction and the egress of HSPCs into circulation [23]. In the same study, low micromolar amounts of Me6TREN dose-dependently inhibited CXCL12-directed *in vitro* migration of Jurkat cells. Me6TREN also increased CXCL12-induced phosphoAKT, phosphoERK and phosphop38 protein levels, although the cAMP pathway was not significantly activated [23]. We did observe neither an inhibitory effect, nor a stimulatory effect of Me6TREN on CXCL12 receptor binding, chemotaxis of Jurkat cells and CXCL12-induced intracellular calcium release (Figs 1C, 2D and 4C). Our findings thus support the idea that Me6TREN most likely inhibits CXCR4 function *in vivo* via an indirect pathway, for instance by upregulating the levels of MMP-9. The peptide analog of CXCL12, CTCE-9908, exhibits anti-tumoral effects in *in vivo* tumor models, including breast, prostate and esophageal cancer, by inhibiting CXCL12-induced primary tumor growth and metastasis [33–36]. Pretreatment of osteosarcoma cells with CTCE-9908 also affects several stages of tumor development *in vitro*, such as adhesion, migration, invasion and growth [34]. In addition, a study by Drenckhan *et al*. showed that CTCE-9908 exhibits anti-proliferative and -migratory effects on esophageal cells [33]. The anti-tumoral effects of CTCE-9908 observed in the above mentioned studies were obtained after long-term (24 hours) treatment with relatively high concentrations of CTCE-9908 (approximately 50 μM), which might explain why we did not observe inhibition of Jurkat cell migration by CTCE-9908 (Fig 4A). Indeed, our experimental design in which all compounds are compared side-by-side under standardized conditions shows that CTCE-9908 likely has rather poor direct anti-CXCR4 activity, if any activity at all. Further studies should be pursued to unravel the mechanism of action of CTCE-9908.

Using computational docking gambogic acid has previously been predicted to physically interact with CXCR4 and this binding is comparable to the binding mode of AMD3100. Experimental data to support this prediction, however, were not included [24]. We demonstrate that gambogic acid competes with CXCL12 for CXCR4 binding, although with relatively low potency (IC_50_ in the micromolar range) when compared to AMD3100. Apart from a presumed direct effect on CXCR4, an indirect inhibitory effect of gambogic acid, driven by its capacity to suppress NF-кB binding to the CXCR4 promoter, was demonstrated. This leads to downregulation of CXCR4 mRNA and protein levels, detectable as early as 12 hours post treatment. Receptor downregulation was correlated with reduced CXCL12-mediated invasion of multiple myeloma cells after 24 hours incubation with gambogic acid [24]. In our study, we found that gambogic acid only weakly inhibited CXCL12-induced calcium signaling and chemotaxis (Figs 2D and 4C). Given the short incubation time of these assays (up to two hours for chemotaxis), this mild inhibitory effect is likely not caused by the decrease in CXCR4 mRNA expression, but rather reflects the weak binding to CXCR4. Taken together, it appears that gambogic acid might modulate CXCR4 function by both direct and indirect mechanisms. However, despite the anti-CXCR4 activity observed for gambogic acid, it is important to note its high cellular cytotoxicity when tested in two different cell lines (*i*.*e*. Jurkat and MT-4 cells) as well as primary cells (*i*.*e*. PBMCs) rendering it a less suitable molecule for use in some preclinical disease models.

WZ811, sharing common structural features with AMD3100, was initially described by Zhan *et al*. [22]. Despite their structural similarity, the binding mode of WZ811 is quite different from that of AMD3100 [37]. WZ811 potently inhibits binding of TN14003 [22], a peptide CXCR4 antagonist [17], but it has been unclear whether WZ811 also directly effects CXCL12 receptor binding. In our experiments WZ811 was unable, even at high concentration, to inhibit the interaction between CXCL12 and CXCR4 on both Jurkat (Fig 1C) as well as SUP-T1 cells (data not shown). Additional binding experiments using labeled CXCR4 monoclonal antibodies targeting distinct receptor regions [*i*.*e*. 12G5 clone (targeting the second extracellular loop) and 1D9 clone (targeting the N-terminal receptor portion)] were performed, but also indicated that WZ811 was unable to reduce mAb binding (data not shown). WZ811 was inactive in all of our pharmacological and functional CXCR4 assays in contrast to previous studies that showed inhibition of CXCL12-induced migration/invasion with even greater potency than AMD3100 [22, 38]. Overall, the lack of inhibitory activity of WZ811 in our diverse set of experiments suggests that WZ811 is not, or at least a very weak CXCR4 inhibitor.

The first generation peptide inhibitor T22 and small molecule antagonist AMD3100 were discovered as potent CXCR4 inhibitors of HIV infection [15, 39]. In order to improve their anti-HIV activity and pharmacokinetic properties, several derivatives and novel agents were designed (*e*.*g*. T140, TC14012, AMD3465, AMD11070 and IT1t) [14, 21]. All of them are potent CXCR4 inhibitors of HIV-1 replication (Table 1). From our comparative study, we conclude that the polyphemusin II-derived CXCR4 peptides exhibit a better activity profile than the tested small molecules, supporting previously published data (Table 1 and S1 Table) [26, 40]. The discrepancy in binding and inhibitory potency between both classes of compounds (*i*.*e*. peptide analogs vs small molecule antagonists) are not clearly understood, but could in part be explained by differences in crucial interaction residues in the receptor’s ligand binding cavity. For instance, crystallization studies of CXCR4 demonstrated that IT1t occupies only part of the binding pocket, whereas the peptide CVX15 (a T140 analog) fills most of the binding pocket volume. Moreover, CVX15 might induce significant conformational changes in the receptor dimer, which could potentially result in negative binding cooperativity of CXCR4 [41].

In summary, we evaluated the binding characteristics and relative potencies of several classes of CXCR4 inhibitors, previously described to inhibit CXCR4 function *in vitro* and/or *in vivo*, in a panel of pharmacological and functional receptor assays. Side-by-side comparison indicated that CTCE-9908, WZ811, Me6TREN and gambogic acid are not, or at least very weak, direct CXCR4 inhibitors, although some of them might modulate CXCR4 function *in vivo* by indirect mechanisms. On the other hand, T22, T140, TC14012, AMD3100, AMD3465, AMD11070 and IT1t demonstrated potent anti-CXCR4 activity, driven by the competition with CXCL12 for receptor occupancy. This makes these latter compounds the best candidate molecules for further evaluation of the role of CXCR4 in (pre-)clinical disease models and drug design.

## Supporting information

S1 Fig Amino acid sequence of the CXCR4-targeting peptides (A) T22, T140, TC14012 and CTCE-9908 and structures of the chemical compounds (B) AMD3100, AMD3465, AMD11070, IT1t and (C) WZ811, Me6TREN, gambogic acid. Disulfide bridges are indicated with a black line.
(TIF)

S2 Fig CXCR4, CXCR7 and CD4 expression on (A) Jurkat, (B) SUP-T1, (C) MT-4 and (D) U87.CD4.CXCR4 cells. **(A), (B) and (C):** Cell surface CXCR4 and CXCR7 expression was quantified by flow cytometry. Red and blue histogram represent the isotype control staining and the CXCR4 or CXCR7 cell surface expression, respectively. **(D):** U87.CD4.CXCR4 cells were stained with mAbs targeting CXCR4, CXCR7, CD4 (blue histogram) and the corresponding isotype controls (red histograms). Cell surface expression of the receptors was continuously monitored throughout all experiments. Data from one representative experiment are shown.
(TIF)

S3 Fig Agonistic activity of the compounds on intracellular calcium flux in U87.CD4.CXCR4 cells. Fluo-2 AM loaded U87.CD4.CXCR4 cells were incubated with test compound for 10 minutes during which changes in intracellular calcium concentration were measured to evaluate the agonistic activity of the compounds in the absence of CXCL12 (time interval: 0–600 seconds). Afterwards, the inhibitory effect of the compounds on the CXCL12-induced calcium flux was determined (time interval: 600–830 seconds). Calcium fluxes are represented as % of baseline (*i*.*e*. mean relative light units in each well during a fixed time interval before compound and CXCL12 addition. For each compound the highest tested concentration is shown: (A) T22, (B) T140, (C) TC14012, (F) AMD3465, (G) AMD11070 and (H) IT1t at 1,000 nM; (E) AMD3100 at 25,000 nM; (K) gambogic acid at 10,000 nM; (D) CTCE-9908, (I) WZ811 and (J) Me6TREN at 100,000 nM. The red and blue line represent the negative (untreated, without CXCL12 stimulation) and positive (untreated, CXCL12 stimulation) control, respectively.
(TIF)

S1 Table Antagonistic activity of CXCR4 inhibitors on CXCL12/CXCR4 binding interaction, CXCL12-induced calcium signaling, chemotaxis and CXCR4 receptor internalization. (DOCX)

## References

[1] Bachelerie F, Ben-Baruch A, Burkhardt AM, Combadiere C, Farber JM, Graham GJ, et al International Union of Basic and Clinical Pharmacology. [corrected]. LXXXIX. Update on the extended family of chemokine receptors and introducing a new nomenclature for atypical chemokine receptors. Pharmacol Rev. 2014;66: 1–79. 10.1124/pr.113.007724 24218476 PMC3880466

[2] Raman D, Sobolik-Delmaire T, Richmond A. Chemokines in health and disease. Exp Cell Res. 2011;317: 575–589. 10.1016/j.yexcr.2011.01.005 21223965 PMC3063402

[3] Vandercappellen J, Van Damme J, Struyf S. The role of CXC chemokines and their receptors in cancer. Cancer Lett. 2008;267: 226–244. 10.1016/j.canlet.2008.04.050 18579287

[4] Balkwill F. The significance of cancer cell expression of the chemokine receptor CXCR4. Semin Cancer Biol. 2004;14: 171–179. 10.1016/j.semcancer.2003.10.003 15246052

[5] Busillo JM, Benovic JL. Regulation of CXCR4 signaling. Biochim Biophys Acta. 2007;1768: 952–963. 10.1016/j.bbamem.2006.11.002 17169327 PMC1952230

[6] Bleul CC, Farzan M, Choe H, Parolin C, Clark-Lewis I, Sodroski J, et al The lymphocyte chemoattractant SDF-1 is a ligand for LESTR/fusin and blocks HIV-1 entry. Nature. 1996;382: 829–833. 10.1038/382829a0 8752280

[7] Deng H, Liu R, Ellmeier W, Choe S, Unutmaz D, Burkhart M, et al Identification of a major co-receptor for primary isolates of HIV-1. Nature. 1996;381: 661–666. 10.1038/381661a0 8649511

[8] Domanska UM, Kruizinga RC, Nagengast WB, Timmer-Bosscha H, Huls G, de Vries EG, et al A review on CXCR4/CXCL12 axis in oncology: no place to hide. Eur J Cancer. 2013;49: 219–230. 10.1016/j.ejca.2012.05.005 22683307

[9] Hernandez PA, Gorlin RJ, Lukens JN, Taniuchi S, Bohinjec J, Francois F, et al Mutations in the chemokine receptor gene CXCR4 are associated with WHIM syndrome, a combined immunodeficiency disease. Nat Genet. 2003;34: 70–74. 10.1038/ng1149 12692554

[10] Nanki T, Hayashida K, El-Gabalawy HS, Suson S, Shi K, Girschick HJ, et al Stromal cell-derived factor-1-CXC chemokine receptor 4 interactions play a central role in CD4+ T cell accumulation in rheumatoid arthritis synovium. J Immunol. 2000;165: 6590–6598. 11086103 10.4049/jimmunol.165.11.6590

[11] Flomenberg N, DiPersio J, Calandra G. Role of CXCR4 chemokine receptor blockade using AMD3100 for mobilization of autologous hematopoietic progenitor cells. Acta Haematol. 2005;114: 198–205. 10.1159/000088410 16269859

[12] Schols D, Struyf S, Van Damme J, Este JA, Henson G, De Clercq E. Inhibition of T-tropic HIV strains by selective antagonization of the chemokine receptor CXCR4. J Exp Med. 1997;186: 1383–1388. 9334378 10.1084/jem.186.8.1383 PMC2199084

[13] Debnath B, Xu S, Grande F, Garofalo A, Neamati N. Small molecule inhibitors of CXCR4. Theranostics. 2013;3: 47–75. 10.7150/thno.5376 23382786 PMC3563081

[14] Zhang H, Kang D, Huang B, Liu N, Zhao F, Zhan P, et al Discovery of non-peptide small molecular CXCR4 antagonists as anti-HIV agents: Recent advances and future opportunities. Eur J Med Chem. 2016;114: 65–78. 10.1016/j.ejmech.2016.02.051 26974376

[15] Murakami T, Nakajima T, Koyanagi Y, Tachibana K, Fujii N, Tamamura H, et al A small molecule CXCR4 inhibitor that blocks T cell line-tropic HIV-1 infection. J Exp Med. 1997;186: 1389–1393. 9334379 10.1084/jem.186.8.1389 PMC2199089

[16] Tamamura H, Xu Y, Hattori T, Zhang X, Arakaki R, Kanbara K, et al A low-molecular-weight inhibitor against the chemokine receptor CXCR4: a strong anti-HIV peptide T140. Biochem Biophys Res Commun. 1998;253: 877–882. 10.1006/bbrc.1998.9871 9918823

[17] Tamamura H, Omagari A, Hiramatsu K, Gotoh K, Kanamoto T, Xu Y, et al Development of specific CXCR4 inhibitors possessing high selectivity indexes as well as complete stability in serum based on an anti-HIV peptide T140. Bioorg Med Chem Lett. 2001;11: 1897–1902. 11459656 10.1016/s0960-894x(01)00323-7

[18] Faber A, Roderburg C, Wein F, Saffrich R, Seckinger A, Horsch K, et al The many facets of SDF-1alpha, CXCR4 agonists and antagonists on hematopoietic progenitor cells. J Biomed Biotechnol. 2007;2007: 26065 10.1155/2007/26065 17541466 PMC1874670

[19] Hatse S, Princen K, De Clercq E, Rosenkilde MM, Schwartz TW, Hernandez-Abad PE, et al AMD3465, a monomacrocyclic CXCR4 antagonist and potent HIV entry inhibitor. Biochem Pharmacol. 2005;70: 752–761. 10.1016/j.bcp.2005.05.035 16011832

[20] Stone ND, Dunaway SB, Flexner C, Tierney C, Calandra GB, Becker S, et al Multiple-dose escalation study of the safety, pharmacokinetics, and biologic activity of oral AMD070, a selective CXCR4 receptor inhibitor, in human subjects. Antimicrob Agents Chemother. 2007;51: 2351–2358. 10.1128/AAC.00013-07 17452489 PMC1913234

[21] Thoma G, Streiff MB, Kovarik J, Glickman F, Wagner T, Beerli C, et al Orally bioavailable isothioureas block function of the chemokine receptor CXCR4 in vitro and in vivo. J Med Chem. 2008;51: 7915–7920. 10.1021/jm801065q 19053768

[22] Zhan W, Liang Z, Zhu A, Kurtkaya S, Shim H, Snyder JP, et al Discovery of small molecule CXCR4 antagonists. J Med Chem. 2007;50: 5655–5664. 10.1021/jm070679i 17958344

[23] Zhang J, Ren X, Shi W, Wang S, Chen H, Zhang B, et al Small molecule Me6TREN mobilizes hematopoietic stem/progenitor cells by activating MMP-9 expression and disrupting SDF-1/CXCR4 axis. Blood. 2014;123: 428–441. 10.1182/blood-2013-04-498535 24196072

[24] Pandey MK, Kale VP, Song C, Sung SS, Sharma AK, Talamo G, et al Gambogic acid inhibits multiple myeloma mediated osteoclastogenesis through suppression of chemokine receptor CXCR4 signaling pathways. Exp Hematol. 2014;42: 883–896. 10.1016/j.exphem.2014.07.261 25034231

[25] Princen K, Hatse S, Vermeire K, De Clercq E, Schols D. Establishment of a novel CCR5 and CXCR4 expressing CD4+ cell line which is highly sensitive to HIV and suitable for high-throughput evaluation of CCR5 and CXCR4 antagonists. Retrovirology. 2004;1: 2 10.1186/1742-4690-1-2 15169555 PMC416571

[26] Hatse S, Princen K, Liekens S, Vermeire K, De Clercq E, Schols D. Fluorescent CXCL12AF647 as a novel probe for nonradioactive CXCL12/CXCR4 cellular interaction studies. Cytometry A. 2004;61: 178–188. 15382150 10.1002/cyto.a.20070

[27] Vermeire K, Princen K, Hatse S, De Clercq E, Dey K, Bell TW, et al CADA, a novel CD4-targeted HIV inhibitor, is synergistic with various anti-HIV drugs in vitro. AIDS. 2004;18: 2115–2125. 15577644 10.1097/00002030-200411050-00003

[28] Mosi RM, Anastassova V, Cox J, Darkes MC, Idzan SR, Labrecque J, et al The molecular pharmacology of AMD11070: an orally bioavailable CXCR4 HIV entry inhibitor. Biochem Pharmacol. 2012;83: 472–479. 10.1016/j.bcp.2011.11.020 22146583

[29] Gravel S, Malouf C, Boulais PE, Berchiche YA, Oishi S, Fujii N, et al The peptidomimetic CXCR4 antagonist TC14012 recruits beta-arrestin to CXCR7: roles of receptor domains. J Biol Chem. 2010;285: 37939–37943. 10.1074/jbc.C110.147470 20956518 PMC2992227

[30] Boerrigter G, Lark MW, Whalen EJ, Soergel DG, Violin JD, Burnett JC Jr. Cardiorenal actions of TRV120027, a novel ss-arrestin-biased ligand at the angiotensin II type I receptor, in healthy and heart failure canines: a novel therapeutic strategy for acute heart failure. Circ Heart Fail. 2011;4: 770–778. 10.1161/CIRCHEARTFAILURE.111.962571 21835984

[31] Magnan R, Escrieut C, Gigoux V, De K, Clerc P, Niu F, et al Distinct CCK-2 receptor conformations associated with beta-arrestin-2 recruitment or phospholipase-C activation revealed by a biased antagonist. J Am Chem Soc. 2013;135: 2560–2573. 10.1021/ja308784w 23323542

[32] Violin JD, Crombie AL, Soergel DG, Lark MW. Biased ligands at G-protein-coupled receptors: promise and progress. Trends Pharmacol Sci. 2014;35: 308–316. 10.1016/j.tips.2014.04.007 24878326

[33] Drenckhan A, Kurschat N, Dohrmann T, Raabe N, Koenig AM, Reichelt U, et al Effective inhibition of metastases and primary tumor growth with CTCE-9908 in esophageal cancer. J Surg Res. 2013;182: 250–256. 10.1016/j.jss.2012.09.035 23117118

[34] Kim SY, Lee CH, Midura BV, Yeung C, Mendoza A, Hong SH, et al Inhibition of the CXCR4/CXCL12 chemokine pathway reduces the development of murine pulmonary metastases. Clin Exp Metastasis. 2008;25: 201–211. 10.1007/s10585-007-9133-3 18071913 PMC2730112

[35] Porvasnik S, Sakamoto N, Kusmartsev S, Eruslanov E, Kim WJ, Cao W, et al Effects of CXCR4 antagonist CTCE-9908 on prostate tumor growth. Prostate. 2009;69: 1460–1469. 10.1002/pros.21008 19588526

[36] Richert MM, Vaidya KS, Mills CN, Wong D, Korz W, Hurst DR, et al Inhibition of CXCR4 by CTCE-9908 inhibits breast cancer metastasis to lung and bone. Oncol Rep. 2009;21: 761–767. 19212637 PMC13435714

[37] Kawatkar SP, Yan M, Gevariya H, Lim MY, Eisold S, Zhu X, et al Computational analysis of the structural mechanism of inhibition of chemokine receptor CXCR4 by small molecule antagonists. Exp Biol Med (Maywood). 2011;236: 844–850. 21697335 10.1258/ebm.2011.010345 PMC3900290

[38] Jung SH, Won KJ, Lee KP, Lee DH, Yu S, Lee DY, et al DJ-1 protein regulates CD3+ T cell migration via overexpression of CXCR4 receptor. Atherosclerosis. 2014;235: 503–509. 10.1016/j.atherosclerosis.2014.05.955 24953490

[39] De Clercq E, Yamamoto N, Pauwels R, Balzarini J, Witvrouw M, De Vreese K, et al Highly potent and selective inhibition of human immunodeficiency virus by the bicyclam derivative JM3100. Antimicrob Agents Chemother. 1994;38: 668–674. 7913308 10.1128/aac.38.4.668 PMC284523

[40] Juarez J, Bradstock KF, Gottlieb DJ, Bendall LJ. Effects of inhibitors of the chemokine receptor CXCR4 on acute lymphoblastic leukemia cells in vitro. Leukemia. 2003;17: 1294–1300. 12835717 10.1038/sj.leu.2402998

[41] Wu B, Chien EY, Mol CD, Fenalti G, Liu W, Katritch V, et al Structures of the CXCR4 chemokine GPCR with small-molecule and cyclic peptide antagonists. Science. 2010;330: 1066–1071. 10.1126/science.1194396 20929726 PMC3074590

