# Supplementary figures and images for: Comparison of cell-based assays for the identification and evaluation of competitive CXCR4 inhibitors
Source: PLoS One. 2017 Apr 14;12(4):e0176057. doi: 10.1371/journal.pone.0176057 (PMC5391968; doi:10.1371/journal.pone.0176057)

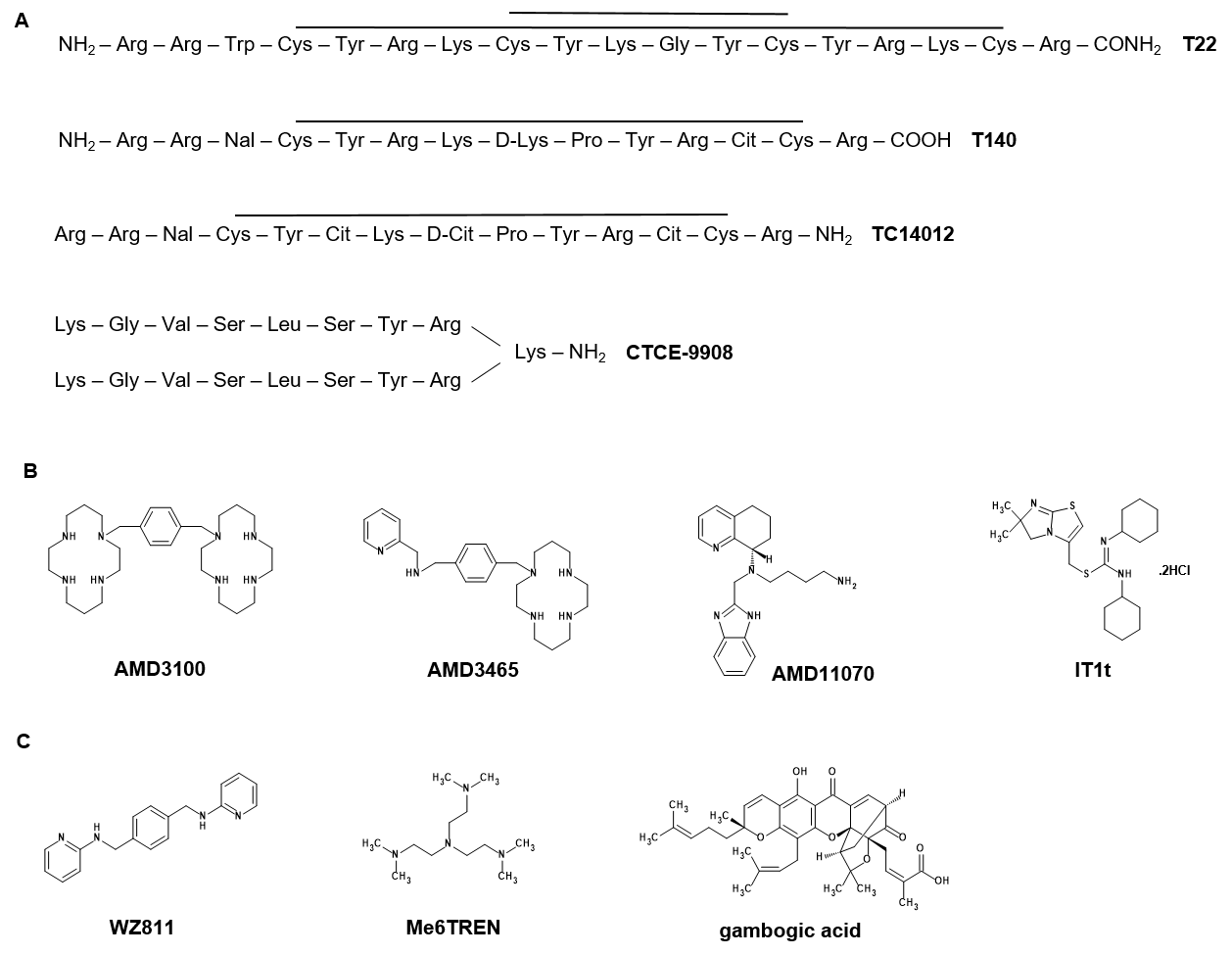

Supplement: S1 Fig — Disulfide bridges are indicated with a black line. (TIF) [file pone.0176057.s001.tif]

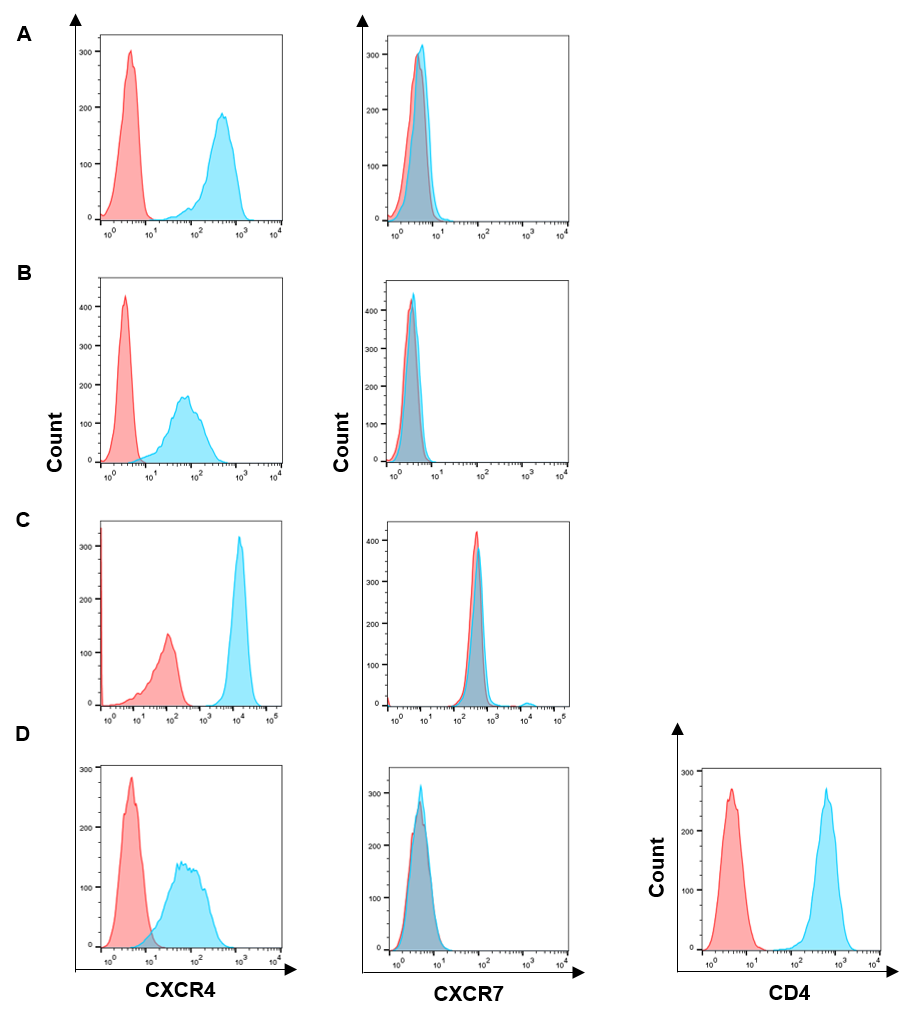

Supplement: S2 Fig — (A), (B) and (C): Cell surface CXCR4 and CXCR7 expression was quantified by flow cytometry. Red and blue histogram represent the isotype control staining and the CXCR4 or CXCR7 cell surface expression, respectively. (D): U87.CD4.CXCR4 cells were stained with mAbs targeting CXCR4, CXCR7, CD4 (blue histogram) and the corresponding isotype controls (red histograms). Cell surface expression of the receptors was continuously monitored throughout all experiments. Data from one representative experiment are shown. (TIF) [file pone.0176057.s002.tif]
