# Supplementary material for: Comparison of cell-based assays for the identification and evaluation of competitive CXCR4 inhibitors
Source: PLoS One. 2017 Apr 14;12(4):e0176057. doi: 10.1371/journal.pone.0176057 (PMC5391968; doi:10.1371/journal.pone.0176057)
Supplement: S1 Table — (DOCX) [file pone.0176057.s004.docx]

|  | IC_50_^a^ (nM) | | | |
| --- | --- | --- | --- | --- |
|  | CXCL12-CXCR4 binding interaction | Calcium signaling | CXCR4 internalization | Chemotaxis |
| T22 | 0.079 ± 0.034 | 9.3 ± 3.2 | 10.2 ± 1.5 | 470.2 ± 16.5 |
| T140 | 0.12 ± 0.025 | 1.2 ± 0.17 | 2.2 ± 0.4 | 73.7 ± 26.0 |
| TC14012 | 0.11 ± 0.0094 | 0.95 ± 0.18 | 1.9 ± 0.2 | 58.3 ± 22.3 |
| CTCE-9908 | 19,145 ± 2,380 | >100,000 | >100,000 | 23,593 ± 1,711 |
| AMD3100 | 12.0 ± 1.1 | 723.0 ± 99.1 | 148.0 ± 36.0 | 1,431 ± 485 |
| AMD3465 | 2.1 ± 0.24 | 53.4 ± 24.3 | 67.3 ± 11.3 | 116.0 ± 15.2 |
| AMD11070 | 0.67 ± 0.10 | 12.3 ± 1.7 | 70.5 ± 13.1 | 74.9 ± 17.7 |
| IT1t | 2.1 ± 0.37 | 23.1 ± 4.6 | 105.7 ± 12.3 | 79.1 ± 9.6 |
| WZ811 | >100,000 | >100,000 | >100,000 | >100,000 |
| Me6TREN | 51,934 ± 8,952 | >100,000 | >100,000 | >100,000 |
| gambogic acid | 7,063 ± 532 | 4,371 ± 672 | >10,000 | 1,888 ± 353 |

^a^ Concentration needed to inhibit CXCL12 receptor binding, CXCL12-induced calcium signaling, chemotaxis or CXCR4 internalization by 50%. Mean IC_50_ ± SEM is shown from two to four independent experiments.
